# Anxiety and Depression in Ireland during COVID-19 – a narrative review

**DOI:** 10.1192/j.eurpsy.2022.210

**Published:** 2022-09-01

**Authors:** V. Sathyanarayanan, D. Shahwar, M. Azeem

**Affiliations:** 1 University College Cork, Medicine, Cork, Ireland; 2 Sidra Medicine, Child And Adolescent Psychiatry, Doha, Qatar

**Keywords:** pandemic, Coronavirus, lockdown, mental health

## Abstract

**Introduction:**

Ireland has been one of the worst affected countries affected by COVID-19 in Europe. Many primary studies from Ireland have documented prevalence of anxiety and depressive disorders during the pandemic and their correlates.

**Objectives:**

To study the prevalence range of anxiety and depression in Ireland, and their correlates during the pandemic.

**Methods:**

We systematically searched Pubmed, PsycInfo and the WHO COVID-19 global research database using key words ( January 2020 - September 2021). We removed duplicates and extracted data into an excel database and carried out a narrative synthesis of the extracted data.

**Results:**

From a total 127 studies, we included 22 studies that met our criteria in our narrative review. Depending on the tool used and the type of population studied, the prevalence of general anxiety disorders varied between 20% and 49.5% while prevalence of depressive disorders ranged between 20.4% and 53.8%. Younger people, health care workers, those who had to give up physical activity, people who had lost income, those who lived alone, infected by COVID-19, or had a higher perceived risk of the disease had a higher prevalence of both anxiety and depression disorders during the pandemic. There was conflicting evidence on prevalence levels among men and women and on whether they had children or not.

**Conclusions:**

COVID-19 has had a profound effect on the mental health of the Irish population. Some population groups are more affected than the others. Addressing mental health concerns of Irish population during and post pandemic should remain as one of the top public health priorities.

**Disclosure:**

No significant relationships.

